# Long-term relationship between everolimus blood concentration and clinical outcomes in Japanese patients with metastatic renal cell carcinoma: a prospective study

**DOI:** 10.1186/s40780-019-0135-5

**Published:** 2019-03-12

**Authors:** Shinya Takasaki, Hiroaki Yamaguchi, Yoshihide Kawasaki, Masafumi Kikuchi, Masaki Tanaka, Akihiro Ito, Nariyasu Mano

**Affiliations:** 1 Department of Pharmaceutical Sciences, Tohoku University Hospital, 1-1 Seiryo-machi, Aobaku, Sendai, Miyagi 980-8574 Japan; 2 Department of Urology, Tohoku University Graduate School of Medicine, 1-1 Seiryo-machi, Aobaku, Sendai, Miyagi 980-8574 Japan

**Keywords:** Everolimus, mTOR, Pharmacokinetics, Renal cell carcinoma, Therapeutic drug monitoring

## Abstract

**Background:**

Everolimus is an oral inhibitor of mammalian target of rapamycin, approved for metastatic renal cell carcinoma (mRCC). Recently, personalized medicine through therapeutic drug monitoring (TDM) is recommended in cancer therapy. In this study, the relationship between everolimus blood concentration and clinical outcomes on a long-term were evaluated in Japanese patients with mRCC.

**Methods:**

Patients with mRCC were enrolled following treatment with everolimus at Tohoku University Hospital between April 2012 and December 2016. The relationship between everolimus trough blood concentration on day 8 of everolimus therapy and just before discontinuation or dose reduction, and their adverse events were evaluated. Patients were divided into two groups based on the median of everolimus blood concentration on day 8 of treatment, and the profiles of adverse events, and efficacy [time to treatment failure (TTF) and progression-free survival (PFS)] were evaluated.

**Results:**

The median (range) everolimus blood concentrations on day 8 after starting everolimus administration and just before discontinuation or dose reduction were 15.3 (8.1–28.0) ng/mL and 14.8 (6.4–58.4) ng/mL, respectively, with no significant difference between these values (*P* = 0.3594). Patients (*n* = 6) with discontinuation or dose reduction following adverse events in everolimus therapy had significantly higher blood concentrations than patients (*n* = 4) with dose maintenance on both day 8 (median, 18.0 vs 8.2 ng/mL; *P* = 0.0139) and just before discontinuation or dose reduction (median, 22.9 vs 9.7 ng/mL; *P* = 0.0142). Median TTF and PFS of the total patients (*n* = 10) were 96 days (95% confidence interval [CI], 26–288) and 235 days (95% CI, 28–291), respectively. Subgroup analysis showed that TTF of the patients with > 15.3 ng/mL (*n* = 5) was not significantly different from that of the patients with ≤15.3 ng/mL (*n* = 5; *P* = 0.5622). Similarly, PFS of the patients with > 15.3 ng/mL was not significantly different from that of the patients with ≤15.3 ng/mL (*P* = 0.3436).

**Conclusions:**

This study demonstrated the long-term relationship between everolimus blood level and clinical outcomes and adverse events in Japanese patients with mRCC. Thus, TDM in everolimus therapy could be a useful tool for the early prediction of adverse events for Japanese patients with mRCC.

## Background

Tyrosine kinase inhibitors and mammalian target of rapamycin inhibitors (mTORi) are molecular targeted drugs for metastatic renal cell carcinoma (mRCC) [1]. Although these targeted drugs of mRCC show higher objective response rate and significantly prolong a median progression-free survival (PFS), various adverse events such as diarrhea, fatigue, vomiting, myelosuppression, and interstitial pneumonia are frequently induced [1]. Recently, personalized medicine for cancer using therapeutic drug monitoring (TDM) is recommended to maximize the efficacy of anticancer drugs, and several evidence of TDM of molecular target drugs such as imatinib and sunitinib have been demonstrated [2, 3].

An mTORi everolimus used for mRCC has already been adapted for TDM in other applications such as the prevention of organ rejection after transplantation [4, 5], and for the treatment of tuberous sclerosis complex [6, 7] and various forms of cancer [8–10]. Everolimus is very effective, but its therapeutic blood concentration range is narrow and the variability of pharmacokinetics among individuals is high. Therefore, it is appropriate to perform individualized medical treatment using TDM [11]. In transplantation settings, trough level of everolimus should be maintained at 3–8 ng/mL when used in combination with other immunosuppressive drugs (calcineurin inhibitor and glucocorticoid) and at 6–10 ng/mL when used without calcineurin inhibitor [11–16]. In the treatment of tuberous sclerosis complex, it is recommended that everolimus concentrations should be managed at 5–15 ng/mL [7, 11, 17]. But, in cancer, there is little evidence of TDM for everolimus in actual clinical practice [11].

Presently, there are several reports on the pharmacokinetics/pharmacodynamics studies of everolimus in cancer [11, 18–20]. Deppenweiler et al. reported that everolimus trough level between 11.9 and 26.3 ng/mL was associated with increase in PFS and decrease in the risk of toxicity [18]. A meta-analysis study by Noguchi et al. demonstrated that the risk of pulmonary adverse events is associated with the administration of everolimus in Japanese patients [19]. Moreover, another meta-analysis study reported the relationship between an increase in everolimus trough level and antitumor effect or risk of high-grade adverse events [20]. However, in cancer patients, there has been no report of monitoring the blood levels of everolimus on a long-term. The dose of everolimus may be reduced following the occurrence of clinically significant hematological or other adverse events. In addition, everolimus blood concentration has been reported to be affected by interaction between drugs [11]. Drugs that alleviate various symptoms will be used for cancer patients with the progress of their symptoms, but these such as antiepileptic drugs may cause drug-drug interactions. That is, in clinical practice, events that may affect everolimus blood concentrations often occur even during everolimus treatment. It is important to evaluate the relationship between everolimus blood level and long-term clinical outcomes. Therefore, in this study, the relationship between everolimus blood concentration and clinical outcomes on a long-term were evaluated in Japanese patients with mRCC.

## Methods

### Patients

The subjects of this study were prospectively recruited from mRCC patients for whom everolimus therapy was scheduled at Tohoku University Hospital from April 2012 to December 2016.

### Chemicals

Everolimus and d4-everolimus as internal standard were purchased from Toronto Research Chemicals (Toronto, ON, Canada). Acetonitrile, methanol, ammonium formate, zinc sulfate, and formic acid were obtained from Wako Pure Chemical Industries (Osaka, Japan). Water was purified using a PURELAB Ultra Genetic system (Organo, Tokyo, Japan).

### Measurement of everolimus blood concentration

The administration schedule of everolimus in this study was in a fasting state. Whole blood samples were obtained just before taking everolimus after day 8 of reaching the steady state of everolimus [21, 22], the sampling was scheduled weekly during hospitalization. For outpatient, the samples were collected for each visit. Everolimus blood concentrations were measured by modifying a previously validated assay [23]. In brief, 100 μL of whole blood sample was mixed with 50 μL of a methanol solution of 100 ng/mL d4-everolimus as an internal standard and preprocessed by 200 μL methanol and 50 μL of 0.2 M zinc sulfate. The samples were centrifuged at 15,000×*g* for 5 min, the supernatants were analyzed by a column-switching liquid chromatography/tandem mass spectrometry system. Analytes were trapped and concentrated at the inlet edge of Shim-pack MAYI-C8 (10 mm × 4.6 mm i.d., 50 μm, GL Sciences, Tokyo, Japan) using the mobile phase [2 mM ammonium formate and 0.1% formic acid in water-methanol (41:9, *v*/v)] at a flow rate 0.5 mL/min. Then, analytes were separated on Luna® phenyl-hexyl column (50 mm × 2 mm i.d., 5 μm, Phenomenex, Torrance, CA, USA) using the mobile phase [2 mM ammonium formate and 0.1% formic acid in water-methanol (1:9, v/v)] at a flow rate 0.2 mL/min. The analysis was performed in selected reaction monitoring mode: *m/z* 975.4 to 542.2 for everolimus; *m/z* 979.5 to 542.2 for d4-everolimus. The quantitative range of everolimus was 1–50 ng/mL. The observed intra-day and inter-day precision and accuracy were below 6.6% and within ±6.8%, respectively. Samples with everolimus blood concentrations higher than the calibration curve range were diluted in saline.

### Evaluation of safety

Adverse events by everolimus therapy were evaluated according to Common Terminology Criteria for Adverse Events version 4.0. The relationship between everolimus blood concentration and everolimus discontinuation or dose reduction due to adverse events was assessed, and everolimus blood concentrations on day 8 and just before discontinuation or dose reduction of everolimus therapy were used for analysis. In addition, the median value of everolimus blood concentration on day 8 was used to classify into two groups, high group and low group, and the association with adverse events was evaluated.

### Evaluation of efficacy

Time to treatment failure (TTF) was defined as the period from the initiation of everolimus therapy to cessation for any cause (including disease progression or adverse events). Progression-free survival (PFS) was defined as the time from the start of everolimus treatment to the objective detection of disease progression or death. Patients were divided into two groups based on the median of everolimus blood concentration on day 8 of treatment, and the efficacy of everolimus (TTF and PFS) was evaluated in the groups.

### Statistical analysis

The cut-off date for this analysis was March 2017. Patients whose blood samples were not obtained after day 8 from the start of everolimus treatment were excluded from the analysis. Continuous variables were compared between two groups by the Wilcoxon rank sum test, and categorical variables were compared by the chi-squared test or Fisher’s exact test. Correlations between everolimus blood concentration on day 8, and age, body surface area (BSA), body mass index (BMI), and estimated glomerular filtration rate (eGFR) were evaluated using Spearman’s rank correlation coefficient. TTF and PFS were estimated using Kaplan-Meier curves and compared using the log-rank test. Differences were considered significant at *P* < 0.05. All statistical analyses were performed using JMP pro 13.1.0 software (SAS Institute Inc., Cary, NC, USA).

## Results

### Patients

**Fig. 1:**
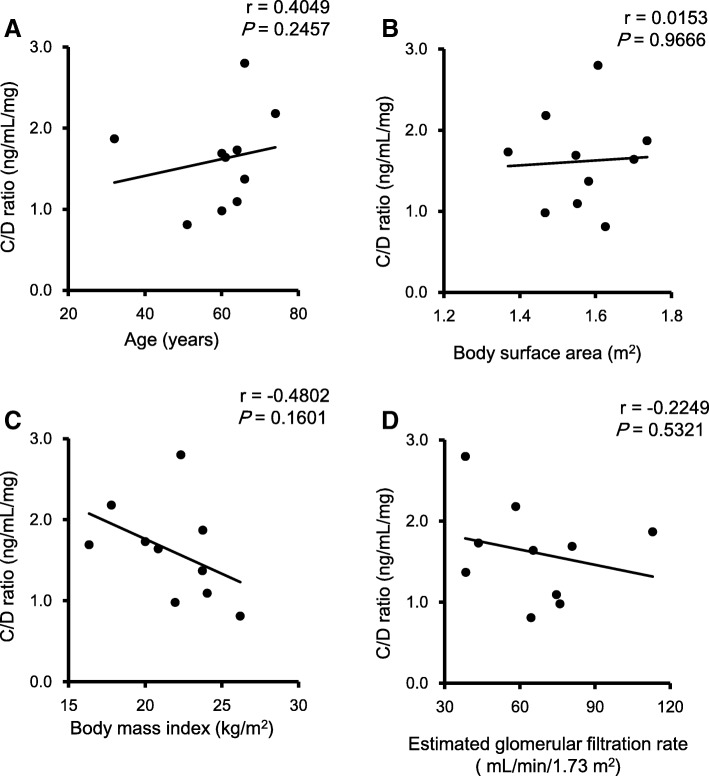
The relationship between the concentration-to-dose (C/D) ratio of everolimus on day 8 and patients’ demographic data. Demographic data include age, body surface area (BSA), body mass index (BMI), and estimated glomerular filtration rate (eGFR) and the relationship was analyzed with Spearman’s rank correlation coefficient

Ten patients with mRCC, who were being administered everolimus, were evaluated in this study. The characteristics of the patients are shown in Table 1. The median (range) everolimus blood concentrations on day 8 after starting everolimus administration and just before discontinuation or dose reduction were 15.3 (8.1–28.0) ng/mL and 14.8 (6.4–58.4) ng/mL, respectively with no significant difference between these values (*P* = 0.3594). Fluctuations in the blood level of everolimus were also observed in some patients. Correlation coefficients between concentration/dose (C/D) and age, BSA, BMI, and eGFR are indicated in Fig. 1. No significant correlation between C/D ratio and each parameter was observed.

**Table 1:** Patients’ characteristics

	Total	Continuation	Discontinuation or dose reduction by adverse events	*P* value
Patients, n	10	4	6	
Age (years)^a^	63 (32–74)	61 (51–64)	65 (32–74)	0.3329 ^b^
Male/Female	5/5	1/3	4/2	0.5238 ^c^
Body weight (kg)^a^	57.7 (46.0–65.8)	58.9 (51.3–63.4)	52.9 (46.0–65.8)	0.4555 ^b^
Body surface area (m^2^)^a^	1.57 (1.37–1.74)	1.59 (1.47–1.70)	1.56 (1.37–1.74)	0.7476 ^b^
Body mass index (kg/m^2^)^a^	22.1 (16.3–26.2)	23.0 (20.9–26.2)	21.2 (16.3–23.8)	0.2410 ^b^
Aspartate aminotransferase (UI/L)^a^	27 (16–43)	29 (17–43)	27 (16–42)	0.6689 ^b^
Alanine aminotransferase (UI/L)^a^	17 (12–47)	26 (12–47)	17 (13–42)	1.0000 ^b^
Serum creatinine (mg/dL)^a^	0.84 (0.61–1.47)	0.68 (0.61–0.92)	0.99 (0.66–1.47)	0.0691 ^b^
eGFR (mL/min/1.73 m^2^)^a^	64.9 (38.2–113.0)	70.0 (64.5–76.0)	50.9 (38.2–113.0)	0.3938 ^b^
ECOG PS, *n*				
0	6	2	4	0.7143 ^c^
1	3	2	1	
2 or more	1	0	1	
Number of prior systemic therapies, *n*				
2	1	1	0	0.3333 ^c^
3	7	2	5	
4 or more	2	1	1	
Initial dose, *n*				
10 mg/day	8	2	6	0.1333^c^
7.5 mg/day	1	1	0	
5 mg/day	1	1	0	
Everolimus blood concentration on day 8 after starting everolimus administration (ng/mL)^a^	15.3 (8.1–28.0)	8.2 (8.1–9.8)	18.0 (13.7–28.0)	0.0139^b^
Everolimus blood concentration just before discontinuation or dose reduction (ng/mL)^a^	14.8 (6.4–58.4)	9.7 (6.4–17.1)	22.9 (12.5–58.4)	0.0142^b^
Change of everolimus blood concentration just before discontinuation or dose reduction from day 8 (absolute value, ng/mL)^a^	1.65 (0.03–36.60)	2.00 (0.03–8.90)	1.40 (0.20–36.60)	0.3374^b^

eGFR: estimated glomerular filtration rate, ECOG PS: Eastern Cooperative Oncology Group Performance Status, ^a^: Values are reported as median (range), ^b^: Continuous variables evaluated by Wilcoxon rank sum test and ^c^: categorical variables by Fisher exact test

### Safety

As shown in Table 1, patients (*n* = 6) with discontinuation or dose reduction by adverse events in everolimus therapy had significantly higher blood concentrations than patients (*n* = 4) with continuation on both the day 8 (median, 18.0 vs 8.2 ng/mL; *P* = 0.0139) and just before discontinuation or dose reduction (median, 22.9 vs 9.7 ng/mL; *P* = 0.0142). The profile of adverse events that occurred in this study is indicated in Table 2, eight patients (80%) had adverse events of all grades and five patients (50%) had adverse events of grade 3 or 4. In addition, we divided the patients into two groups (low level group, ≤ 15.3 ng/mL and high level group, > 15.3 ng/mL) on the basis of the blood concentration of everolimus on day 8 using the median value, and the safety of the drug was evaluated in the two groups of patients. In the low level group (*n* = 5), patients with adverse events of all grades were 3 (60%) and those with adverse events of grade 3 or 4 were 2 (40%). In the high level group (n = 5) of everolimus, patients with adverse events of all grade were 5 (100%) and those with adverse events of grade 3 or 4 were 3 (60%). For the grade 3 or 4 adverse events, pneumonitis and leukopenia were confirmed in two patients, one from the low level group and the other from the high level group. In the high level group, grade 3 hyperglycemia, hypoalbuminemia, and increased γ-glutamyltransferase were observed in one patient, which we have previously reported [24]. Table 3 shows the mean value ± standard deviation (SD) of everolimus blood concentration for each patient, everolimus blood concentration at the time of discontinuation or dose reduction, and the adverse events that caused discontinuation or dose reduction.

**Table 2:** Relationship between adverse events and everolimus blood concentration

	Total (n = 10)		Everolimus blood concentration just before discontinuation or dose reduction (ng/mL)			
			≤ 15.3 (n = 5)		> 15.3 (n = 5)	
	All grades	Grade 3 or 4	All grades	Grade 3 or 4	All grades	Grade 3 or 4
Number of patients						
Any event	8	5	3	2	5	3
Fatigue	1	0	0	0	1	0
Nausea	1	0	0	0	1	0
Vomiting	1	0	0	0	1	0
Mucosal inflammation	5	0	2	0	3	0
Diarrhea	1	0	0	0	1	0
Rash	2	0	1	0	1	0
Pneumonitis	4	2	2	1	2	1
Increased aspartate aminotransferase	2	0	0	0	2	0
Increased alanine transaminase	2	0	0	0	2	0
Increased alkaline phosphatase	3	0	0	0	3	0
Increased γ-glutamyltransferase	1	1	0	0	1	1
Leukopenia	3	2	1	1	2	1
Neutropenia	2	0	1	0	1	0
Thrombocytopenia	2	0	0	0	2	0
Anemia	2	0	0	0	2	0
Hypoalbuminemia	1	1	0	0	1	1
Hyperglycemia	2	1	0	0	2	1

**Table 3:** Everolimus blood concentration at the time of discontinuation or dose reduction by adverse events

Patient number	Number of measurements	Everolimus blood concentration, Mean ± SD (ng/mL)	Discontinuation or dose reduction by adverse events			
			Discontinuation or dose reduction	Date (day)	Everolimus blood concentration (ng/mL)	Adverse events
Pat.1	22	11.2 ± 4.6				
Pat.2	9	16.4 ± 6.2	Discontinuation	147	17.5	AST (G2), ALT (G2), ALP (G2), Hyperglycemia (G2), Mucosal inflammation (G2), Fatigue (G2),
						Pneumonitis (G1), Diarrhea (G1), Leukopenia (G1), Neutropenia (G1)
Pat.3	2	13.1 ± 0.6	Discontinuation	26	12.5	Pneumonitis (G3), Mucosal inflammation (G2)
Pat.4	4	27.9 ± 18.0	Dose reduction	15	58.4	Hyperglycemia (G3), Hypoalbuminemia (G3), γ-GTP (G3), AST (G2), ALP (G2), ALP (G1)
			Discontinuation	41	19.1	AST (G1), ALT(G1), ALP(G1)
Pat.5	15	8.4 ± 2.5				
Pat.6	3	10.2 ± 0.6				
Pat.7	12	18.8 ± 4.8	Discontinuation	265	20.4	Leukopenia (G3), Thrombocytopenia (G2)
Pat.8	3	8.2 ± 0.5				
Pat.9	4	27.6 ± 4.9	Discontinuation	98	35.4	Pneumonitis (G3), Mucosal inflammation (G2)
Pat.10	5	18.7 ± 1.3	Dose reduction	16	17.1	Mucosal inflammation (G2), ALP (G1), Nausea (G1), Vomiting (G1)
			Discontinuation	93	19.5	ALP (G1), Nausea (G1)

AST: Increased aspartate aminotransferase, ALT: Increased alanine transaminase, ALP: Increased alkaline phosphatase, γ-GTP: Increased γ-glutamyltransferase

### Efficacy

**Fig. 2:**
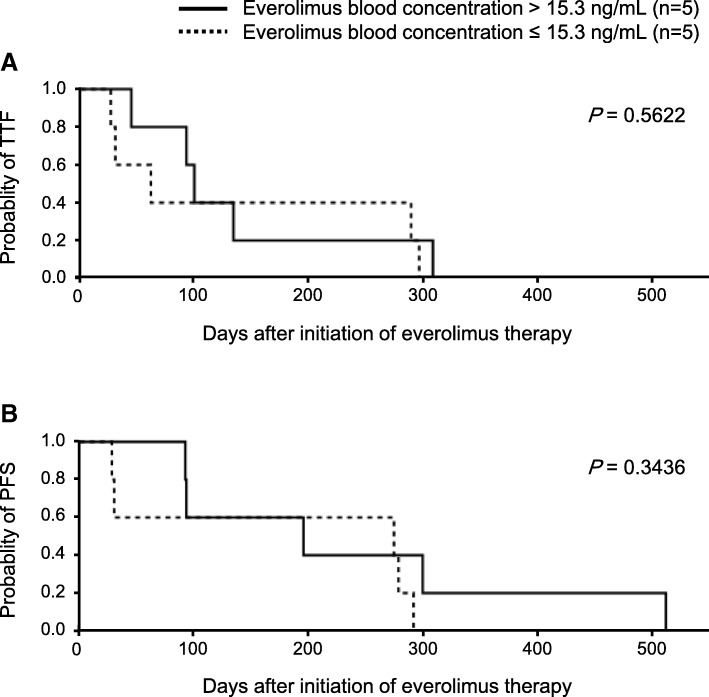
The relationships between everolimus blood concentration and efficacy. Efficacy was evaluated as time to treatment failure (TTF) (A) and progression-free survival (PFS) (B) with the Kaplan-Meier method and the log-rank test

Median TTF and PFS of all the patients (*n* = 10) were 96 days [95% confidence interval (CI), 26–288] and 235 days (95% CI, 28–291). Subgroup analysis using median value (15.3 ng/mL) of everolimus blood concentration on day 8 showed that the TTF of patients with > 15.3 ng/mL (*n* = 5) was not significantly different from that of patients with ≤15.3 ng/mL (n = 5; *P* = 0.5622; Fig. 2a). Similarly, the PFS of patients with > 15.3 ng/mL was not significantly different from that of patients with ≤15.3 ng/mL (*P* = 0.3436; Fig. 2b).

### Clinical application to measurement of everolimus blood concentration

**Fig. 3:**
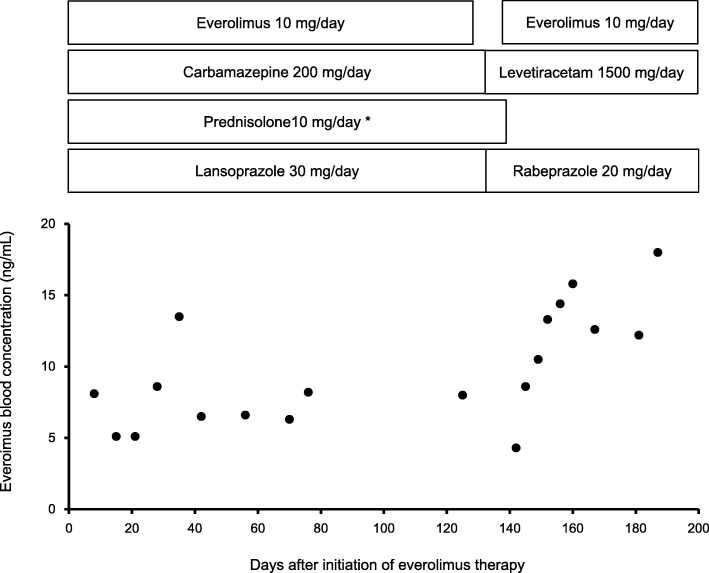
Changes in blood concentration of everolimus before and after combination with concomitant drugs (carbamazepine, prednisolone, and lansoprazole). *Prednisolone was reduced from 10 mg/day to 5 mg/day at the same time as carbamazepine and lansoprazole termination, and was discontinued after 1 week

A case of drug-drug interaction detected by the measuring of blood concentration of everolimus is indicated in Fig. 3. Pat.1 in Table 3 is a 52-year-old Japanese female diagnosed with cell carcinoma 5 years ago. She underwent a partial right nephrectomy for clear cell carcinoma and the following year, her lung metastasis was discovered and sequentially treated with interferon and sunitinib. The sunitinib therapy was changed to everolimus when she was diagnosed with brain metastasis. The patient was administered carbamazepine for neurologic symptoms and prednisolone for cerebral edema associated with brain metastasis. Other concomitant medications were lansoprazole, domperidone, rebamipide, sodium ferrous citrate, and probucol. There were few adverse events of grade 2 or more after the initiation of everolimus 10 mg. The average trough concentration of everolimus in concomitant medications at the start of everolimus was 7.3 ng/mL in patients, whereas the mean level of patients treated with 10 mg of everolimus in a clinical trial was 13.2 ng/mL [22]. Therefore, administration of carbamazepine, prednisolone, and lansoprazole was discontinued because of its ability to induce cytochrome P450 (CYP) 3A4 [25–27]—the main metabolic enzyme of everolimus [11]. Considering less interaction with CYP3A4, carbamazepine was switched to levetiracetam [28], lansoprazole was changed to rabeprazole [29], and prednisolone was stopped after dose reduction. After discontinuing these drugs (carbamazepine, prednisolone, and lansoprazole), the blood concentration of everolimus gradually increased. There were no serious adverse events and no significant change in liver and kidney function during this everolimus treatment, and everolimus therapy lasted for half a year.

## Discussion

In this study, everolimus blood levels of the patients with discontinuation or dose reduction by adverse events were significantly higher than the patients with continuation (Table 1). Deppenweiler et al. reported that everolimus trough levels higher than 26.3 ng/mL were associated with increased risk of adverse events [18]. In the patients (Pat.2, Pat.4, Pat.7, Pat.9, and Pat.10) who exceeded the average everolimus blood level of 16.4 ng/mL, there was discontinuation or dose reduction in the everolimus therapy due to adverse events (Table 3). Everolimus treatment was discontinued in Pat.3 due to grade 3 pneumonitis even though the everolimus level was 13.1 ng/mL, which was not higher than that of other patients (Table 3). Subsequently, Pat.3 was diagnosed with interstitial pneumonia and because symptoms might continue to develop in the patient, steroid pulse therapy was required. The toxic range of interstitial pneumonia by everolimus may be lower than other adverse events, therefore it is better to increase the number of cases and verify in the future. In many cases, TDM of everolimus is considered useful in predicting the occurrence of adverse events.

In this study, there was no significant difference between the median blood everolimus concentration on day 8 (15.3 ng/mL) and just before discontinuation or dose reduction of that therapy (14.8 ng/mL). These values were almost equal to the mean trough value 15.99 ng/mL [19] and 15.65 ng/mL [20] in previous reports. However, the everolimus levels fluctuated largely in Pat 4 (21.8 to 58.4 ng/mL) and Pat.9 (28.0 to 35.4 ng/mL). They had serious adverse events leading to dose reduction and discontinuation. In addition, Pat.1 had fluctuations in everolimus levels due to drug-drug interaction (Fig. 3). In cancer treatment, various supportive therapies are used, and this may cause drug-drug interaction. For instance, antiepileptic drugs are sometimes used for symptomatic relief, but because of many interactions that can occur between drugs, caution is needed in the administration anticancer drugs [11]. Hence, since intra-individual variations in everolimus pharmacokinetics are large and it is affected by concomitant drugs or food components, routine TDM may be effective for everolimus therapy [11]. In addition, large inter-individual variations were also observed in this study (Fig. 1 and Table 3). It is known that the pharmacokinetics of everolimus is affected by drugs and food, as well as intra-individual [11]. To date, there is not sufficient clinical evidence that inter-individual its differences in metabolic enzymes and transporters affect everolimus pharmacokinetics [11].

Ravaud et al. [20] and Deppenweiler et al. [18] reported that everolimus blood level was directly correlated with the antitumor effect, but in this study, there was no significant difference between the TTF and PFS of the high everolimus level group and those of the low everolimus level group (Fig. 2). However, there were some differences between this study and the previous ones. The reports of Ravaud et al. [20] are based on the results of a phase II and III clinical trials, but our patients had worse performance status and more prior systemic therapies than those of the trial. In the research of Deppenweiler et al., the diagnosis of the patients was mainly breast cancer (*n* = 42, 77.8%) and few patients with kidney cancer (*n* = 10, 18.5%) [18], and the relationship between everolimus blood level and antitumor effect may differ depending on the type of cancer. In addition, our study involved only Japanese patients who were also less in number than in the previous studies.

The limitation of the present study was that, it was a small case study and unlike clinical trials, patients with poor performance status or many prior systemic therapies made it difficult to evaluate efficacy. Further studies on the pharmacokinetics/pharmacodynamics of everolimus are required to determine the clinical utility of TDM in oncology settings. Moreover, it is necessary to evaluate the significance of everolimus TDM by a randomized comparative study between TDM group and non-TDM group. This information would help to maximize the therapeutic potential of everolimus TDM for cancer while minimizing severe adverse events.

## Conclusions

The present study demonstrated the long-term relationship between everolimus blood level and clinical outcomes and it showed that everolimus blood level correlate with adverse events in Japanese patients with mRCC. The relation with efficacy was not sufficiently evaluated due to the small number of cases in this study. It is necessary to study further in the future. Consequently, TDM in everolimus therapy could be a useful tool for the early prediction of adverse events in Japanese patients with mRCC.

## Abbreviations

BMI: Body mass index
BSA: Body surface area
C/D: concentration-to-dose
CI: confidence interval
eGFR: estimated glomerular filtration rate
mRCC: Metastatic renal cell carcinoma
mTORi: Mammalian target of rapamycin inhibitor
PFS: Progression-free survival
SD: Standard deviation
TDM: Therapeutic drug monitoring
TTF: Time to treatment failure
